# Effect of Ball-Milling Treatment Combined with Glycosylation on the Structure and Functional Properties of *Litopenaeus vannamei* Protein

**DOI:** 10.3390/foods13091284

**Published:** 2024-04-23

**Authors:** Dan Wang, Yangliu Liu, Mingzhu Guo, Jilu Sun

**Affiliations:** College of Food Science and Technology, Hebei Agricultural University, Baoding 071001, China; wd03080201@163.com (D.W.); lylhebei@163.com (Y.L.); gmz523225334@hebau.edu.cn (M.G.)

**Keywords:** *Litopenaeus vannamei* protein, ball milling, glycosylation, structure, functional property

## Abstract

*Litopenaeus vannamei* protein (LVP) is a high-quality protein. However, its functional properties do not fully meet the needs of food processing. In this study, LVP-xylose conjugates were prepared by conventional wet heat method (GLVP) and ball-milling-assisted wet heat method (GBLVP), respectively. The changes in structure and functional properties of the glycosylated LVP were explored. The findings revealed that ball-milling pretreatment increased the grafting degree to 35.21%. GBLVP had a sparser surface structure and lower particle size than GLVP. FTIR spectra showed that xylose was grafted onto LVP successfully and GBLVP had the lowest α-helix content. Compared with GLVP, GBLVP had a decrease in intrinsic fluorescence intensity and surface hydrophobicity, and an increase in UV absorption intensity. Moreover, GBLVP had higher foaming capacity, solubility and water-holding capacity, and lower allergenicity than GLVP. However, ball-milling pretreatment had a negative impact on the vitro digestibility and oil-holding capacity of GBLVP. In conclusion, ball-milling-assisted treatment of glycosylation could effectively improve the functional properties of LVP, benefiting the broader application of LVP in the food industry.

## 1. Introduction

*Litopenaeus vannamei* is one of the three most farmed shrimp species [1]. It is a popular seafood due to its richness in high-quality protein, essential amino acids, unsaturated fatty acids, and important minerals [2]. As protein resources are continuously developed and utilized, the nutritional value and commercial importance of marine proteins are attracting more attention [3]. *Litopenaeus vannamei* protein (LVP) is a potential source of functional protein. However, the functional properties of natural proteins are usually not fully adequate for food production [4]. Therefore, it is necessary to develop efficient modification methods to improve the processability of natural proteins and broaden their applications.

Protein glycosylation involves chemical interactions between amino groups of proteins and carbonyl groups of reducing sugars. Glycation methods include dry heat method and wet heat method [5]. This reaction can take place in a gentle and safe environment, and is widely used to alter protein structures and improve the functional properties of proteins, such as emulsibility, solubility, foaming properties, antimicrobial activity and antioxidant activity [6]. In industrial production, the wet heat method is superior to the dry heat method because it allows complete contact between the reactants, thereby accelerating the reaction rate [7]. However, the wet heat method may have some disadvantages, such as incomplete reaction, low grafting degree, complex products and complicated control [8].

Some physical techniques can compensate for the limitations of the traditional glycosylation method, resulting in shortening the glycosylation time, increasing the degree of grafting and further improving functional properties of the glycosylated products [9]. Common physically assisted methods include ultrasonication, microwave treatment, high-pressure homogenization and ball-milling treatment. The ball milling treatment utilizes collision and friction forces to decrease the particle size of proteins, thus changing their processing characteristics [10]. This technique has the advantages of high efficiency, environmental friendliness and safety [11], making it widely utilized in protein modification. Simultaneously, Wu et al. demonstrated that the ball-milling-assisted method enhances the degree of grafting in glycosylated products, along with their foaming, water retention and gelling properties, compared to single glycosylation [12].

So far, some research has been carried out on the modification of LVP. Recent studies have shown that utilizing ultrasound [13] and ultrahigh pressure [14] can enhance the gel properties of shrimp myofibrillar proteins. Dong et al. utilized microwaves to decrease the allergenicity of shrimp protein and enhance its solubility [15]. Furthermore, Duppeti et al. discovered that boiling and microwave drying improved the emulsification properties of shrimp protein, whereas hot air drying and roasting greatly enhanced its foaming properties [16]. Obviously, that research primarily focused on the single modification approach. As for the combined modification methods, the relevant research is still rarely reported. Long et al. demonstrated that the high-pressure combined heating treatment effectively mitigated the allergenicity of shrimp tropomyosin [17]. Zhang et al. highlighted the superiority of ultrasound-microwave combined treatment over singular methods, indicating that this approach could reduce the particle size of myofibrillar proteins and enhance their gel properties [18]. So, it is necessary to further develop more effective methods.

However, the promising approach of ball-milling-assisted glycosylation has not been reported for the modification of LVP. Therefore, this study aimed to examine the influence of ball-milling treatment combined with wet glycosylation on the structure and functional properties of LVP. This research might offer novel insights about LVP modification and theoretical guidance for improving the functionality of LVP, which would be helpful for widening the application of LVP in the food industry.

## 2. Materials and Methods

### 2.1. Materials

The shrimp (*Litopenaeus vannamei*) were purchased from the Agricultural University Technology Market (Baoding, China). The average length and weight of the shrimp were 13.88 ± 0.74 cm, 15.35 ± 1.52 g, respectively. Xylose, KBr, Tris, O-phthalaldehyde (OPA), bromophenol blue (BPB) and BCA protein concentration determination kit were purchased from Solarbio Co., Ltd. (Beijing, China). All chemicals were of analytical grade.

### 2.2. Methods

#### 2.2.1. Preparation of LVP

Following the decapitation, peeling and deveining of the shrimp, the shrimp meat was pulverized and homogenized with distilled water in the 1:3 (*w*/*v*) ratio using a Joyoung juicer (Joyoung Co., Jinan, China). The resulting homogenate was then adjusted to pH 11.5 using NaOH (2 mol/L) to solubilize the protein in the shrimp. The clarified protein solution was obtained by centrifuging at 10,173× *g* for 15 min at 4 °C. After adding HCl (2 mol/L) to the supernatant to lower its pH to 5.5, the protein precipitation was obtained by centrifugation under the same conditions. Finally, the protein precipitate was adjusted to pH 7.0 after redissolving with water, and lyophilized to form LVP (91.44 ± 1.63%, N × 6.25).

#### 2.2.2. Ball-Milling Treatment of LVP

Two 2.0 cm diameter, two 1.5 cm diameter and four 0.5 cm diameter stainless steel grinding beads were placed in a 50 mL grinding jar together with LVP (6 g). The LVP was processed in a ball mill (QM-100S, Wuzhou Dingchuang Technology Co., Ltd., Beijing, China) at 20 Hz for 10 min to obtain ball-milled *Litopenaeus vannamei* protein (BLVP). 

#### 2.2.3. Glycosylation of LVP and BLVP

**Figure 1:**
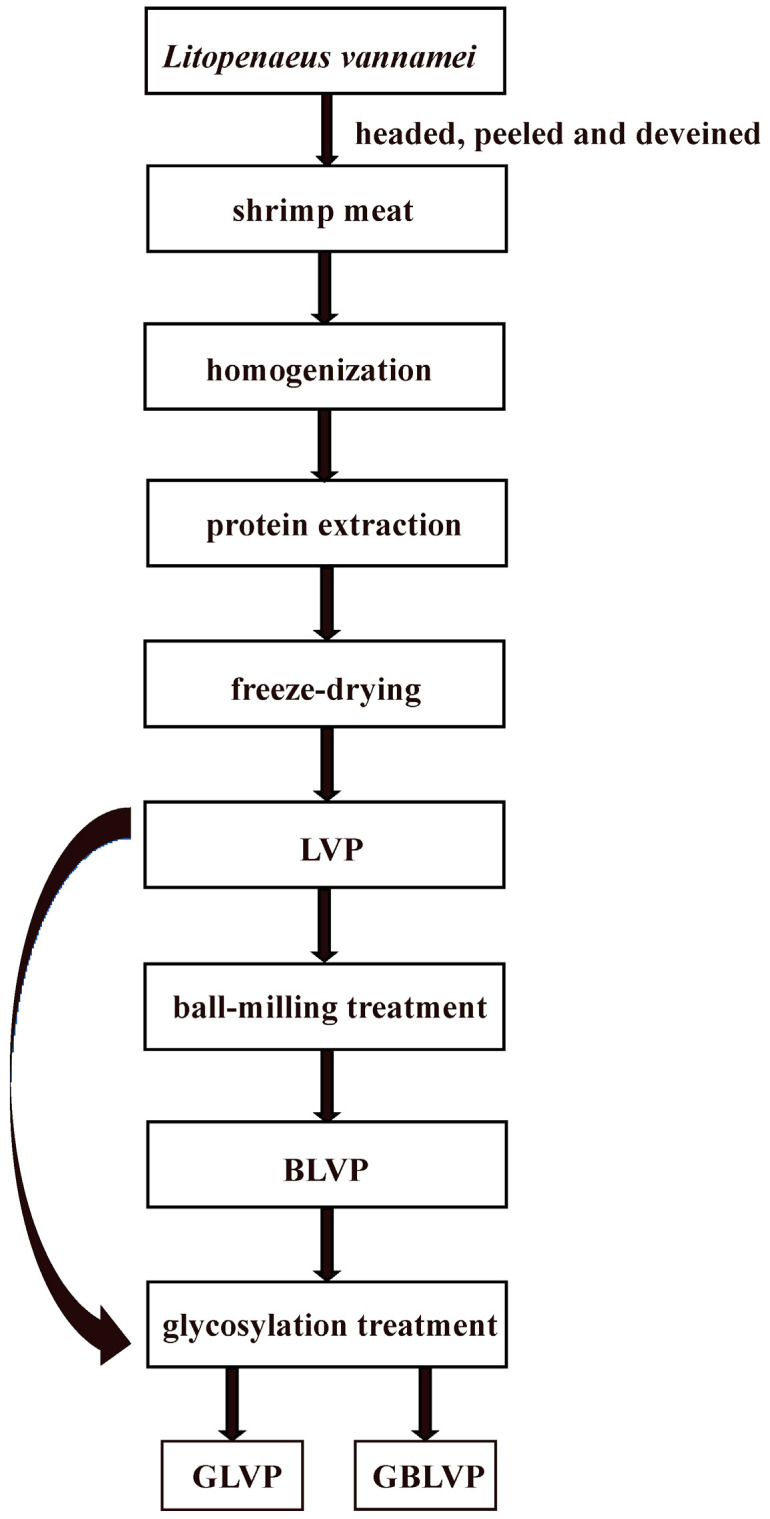
Flow chart for the preparation and modification of LVP.

According to the method of Wu et al. [12] with minor adjustments, LVP and BLVP were prepared as a 5% (*w*/*v*) solution with PBS (0.05 M, pH 7.6), respectively. After being stirred for 1 h, the solution was incubated at 85 °C for 75 min at a protein-to-xylose ratio of 1:2 (*w*/*w*). The reaction solution was promptly transferred to ice-cold water to terminate the reaction. To remove the unbound xylose, the mixture was continuously dialyzed at 4 °C for 24 h. After dialysis, the glycosylated LVP (GLVP) or glycosylated BLVP (GBLVP) were obtained by freeze-drying the mixtures. The process of the preparation and modification of LVP are shown in Figure 1. 

#### 2.2.4. Measurement of Degree of Grafting

The degree of grafting (DG) was determined by OPA method [19]. Briefly, 4 mL of OPA reagent was vortex-mixed with 200 μL of the sample solution (2 mg/mL) prepared from distilled water and incubated at 35 °C for 2 min. The absorbance of the reaction solution was measured at 340 nm. The OPA reagent was obtained by mixing 40 mg OPA, 1 mL methanol, 2.5 mL 20% (*w*/*v*) SDS and 25 mL of 0.1 mol/L borax solution with 100 μL of β-mercaptoethanol and capacitating in a 50 mL volumetric flask with distilled water.

(1) $$DG\;\left(\%\right)=\frac{A_{0}-A_{1}}{A_{0}}$$

where A_0_ and A_1_ represent sample absorbance before and after glycosylation, respectively.

#### 2.2.5. Scanning Electron Microscope Observation

The samples were uniformly adhered to the observation stage and observed by a scanning electron microscope (SEM) (Zeiss Sigma 300, Carl Zeiss AG, Berlin, Germany) after being sprayed with gold. Moreover, the observation was carried out at 300× magnification.

#### 2.2.6. Measurement of Particle Size Distribution

Samples were pretreated following the method originally developed by Yu et al. [20]. Sample solution (0.1 g/mL) prepared from distilled water was stirred for 1 h and centrifuged for 2 min (25 °C, 1030× *g*). The obtained supernatant was subjected to a Nano Particle Sizer (ZS90, Malvern Instruments Ltd., Worcestershire, UK) to record its particle size distribution.

#### 2.2.7. Analysis of Protein Secondary Structure

Secondary structure was examined using the approach of Hadidi et al. [21]. The sample (2 mg) and KBr (150 mg) were ground well and pressed into thin slices. The sheets were scanned with a Fourier transform infrared (FTIR) spectrometer (IRAffinity-IS, Shimadzu Ltd., Kyoto, Japan) over the range of 400–4000 cm^−1^ for 64 scans at a resolution of 4 cm^−1^. The detailed contents of the secondary structure components were calculated by baseline correction, deconvolution, second derivative processing and Gaussian fitting according to the Peak Fit 4.12 software.

#### 2.2.8. Measurement of Surface Hydrophobicity

The surface hydrophobicity (H_0_) was determined according to the method described by Chelh et al. [22] In the treatment group, 0.2 mL of BPB solution (1 mg/mL) was mixed with 1 mL of sample solution (5 mg/mL) prepared from distilled water. In the blank, the sample solution was substituted with distilled water. They were centrifuged (25 °C, 4167× *g*, 15 min) after protection from light for 10 min. The absorbances A_1_ and A_0_ of the treatment and blank were measured at 595 nm.

(2) $$H_{0}\;\left(\mu g\right)=\frac{A_{0}-A_{1}}{A_{0}}\times 200$$

#### 2.2.9. Fluorescence Spectroscopy Analysis

Sample solution (5 mg/mL) prepared from distilled water was diluted 10-fold and then transferred into a cuvette to be analyzed on fluorescence spectrophotometry (F-320, Tianjin GangDong Scientific Co., Ltd., Tianjin, China). The instrument was set at 280 nm for the excitation wavelength, 5 nm for the slit width and 300–400 nm for the recorded spectral values.

#### 2.2.10. UV Full-Wavelength Scanning

The UV spectra of the sample solution (0.1 mg/mL) prepared from distilled water were measured using a UV spectrophotometer (UV-2800A, Unico Shanghai Analysis Co., Shanghai, China) in the range of 250~400 nm, allowing for the observation of changes in the absorption peak.

#### 2.2.11. Measurement of Solubility

The solubility was determined following the methodology of Amiratashani et al. [23], with minor adjustments. The samples were dissolved in 0.1 mol/L of PBS solution (pH 7.0) to prepare a 2 mg/mL solution. After centrifugation at 6511× *g* for 10 min at 4 °C, the obtained supernatant protein and total protein concentration were measured using the BCA and Kjeldahl methods, respectively. The final solubility was calculated using Equation (2).

(3) $$Solubility\;\left(\%\right)=\frac{Supernatant\;protein\;content}{total\;protein\;content}\times 100\%$$

#### 2.2.12. Measurement of Foaming Capacity and Foaming Stability

The foaming capacity (FC) and foaming stability (FS) were determined following a previous method with minor modifications [24]. A sample solution of 20 mg/mL was prepared in distilled water and thoroughly mixed. After that, 30 mL of the sample solution was sheared with a high-speed homogenizer (FSH-2, Changzhou Guohua Electric Co., Changzhou, China) at 10,000 r/min for 1 min. The foaming solution was immediately poured into a measuring cylinder and the volume (V_0_) was measured. The volume of the solution remaining after 10 min of standing (V_10_) was also recorded. The formulas used for the calculation of FC and FS are as follows:

(4) $$FC\;\left(\%\right)=\frac{V_{0}-30}{30}\times 100\%$$

(5) $$FS\;\left(\%\right)=\frac{V_{10}-30}{V_{0}-30}\times 100\%$$

#### 2.2.13. Measurement of Water-Holding Capacity and Oil-Holding Capacity

The water-holding capacity (WHC) and oil-holding capacity (OHC) were measured following the approach of Ortega et al. [25] with minor adjustments. After vortexing the mixture (5 mL of water or oil and 0.1 g of samples) for 1 min, the mixture was allowed to stand at 25 °C for 1 h. Subsequently, the supernatant was poured out after centrifuging at a speed of 4167× *g* for 15 min at 25 °C. The formulas are as follows:

(6) $$WHC\;\left(\%\right)=\frac{M_{1}-M_{0}}{M_{0}}\times 100\%$$

(7) $$OHC\;\left(\%\right)=\frac{M_{2}-M_{0}}{M_{0}}\times 100\%$$

where, M_0_, M_1_ and M_2_ represent the weight of the sample, aqueous sediment and oily sediment, respectively.

#### 2.2.14. Measurement of In Vitro Digestibility

The in vitro digestibility was determined using established methods outlined by Dong et al. [26]. The sample solution (3 mg/mL) prepared with distilled water was adjusted to pH 1.5 using 1 mol/L of HCl solution, followed by mixing the prepared sample solution with pepsin (5 mg/mL) at a ratio of 100:1. The first stage of digestion was conducted for 2 h at 37 °C. Then, this digestion was terminated by adding 1 mol/L of NaOH solution to pH 7.8. For the second stage of digestion, trypsin solution (10 mg/mL) was added at a ratio of 1:30 of enzyme to substrate, which continued for an additional two hours at 37 °C. After digestion, the enzymes were inactivated through boiling. The BCA method was used to determine the protein content of the digested samples. The standard curve of BCA method was constructed by bovine serum protein. Kjeldahl method was used to determine the protein concentrations of samples before digestion. The formula is as follows:

(8) $$Digestibility\;\left(\%\right)=\frac{C_{1}-C_{2}\times D}{C_{1}}\;$$

where C_1_ and C_2_ are the protein concentrations before and after digestion, respectively. D is the dilution number.

#### 2.2.15. Measurement of Allergenicity 

The tropomyosin (TM) content in the samples was determined using a double antibody sandwich ELISA kit (Shanghai MLBIO Biotechnology Co., Ltd., Shanghai, China). The ELISA kit employed a rabbit anti-shrimp TM antibody as the primary antibody and a HRP labeled rabbit anti-TM antibody as detection antibody. The limits of detection (LOD) and quantification (LOQ) were 0.1 ng/mL and 1.5 ng/mL, respectively. The procedure of the kit was as follows: Except for the blank wells, the standard and sample wells of the antibody-coated microtiter plate were sequentially added with different concentrations of standard solution and sample solution (1 mg/mL), respectively. The standard and sample wells were mixed with the enzyme conjugate and incubated for 60 min at 37 °C. After incubation, the wells were washed and dried five times with washing solution. Subsequently, the reaction was carried out at 37 °C for 15 min with the addition of the color-developing solution. Finally, a termination solution was added to each well and the absorbance was measured at 450 nm.

### 2.3. Statistical Analysis

Each treatment included three samples, and all the experiment was performed in triplicate. The results are presented as the mean ± standard deviation (Mean ± SD). One-way analysis of variance (ANOVA) and Duncan and LSD multiple range tests were performed using SPSS software (version 26.0, SPSS Institute Inc., Chicago, IL, USA) to determine significant differences. A significance level of *p* < 0.05 was adopted. Images were generated using Origin software (version 2020, Origin Lab, Inc., Northampton, MA, USA).

## 3. Results and Discussion

### 3.1. Effect on the Degree of Grafting of LVP

**Figure 2:**
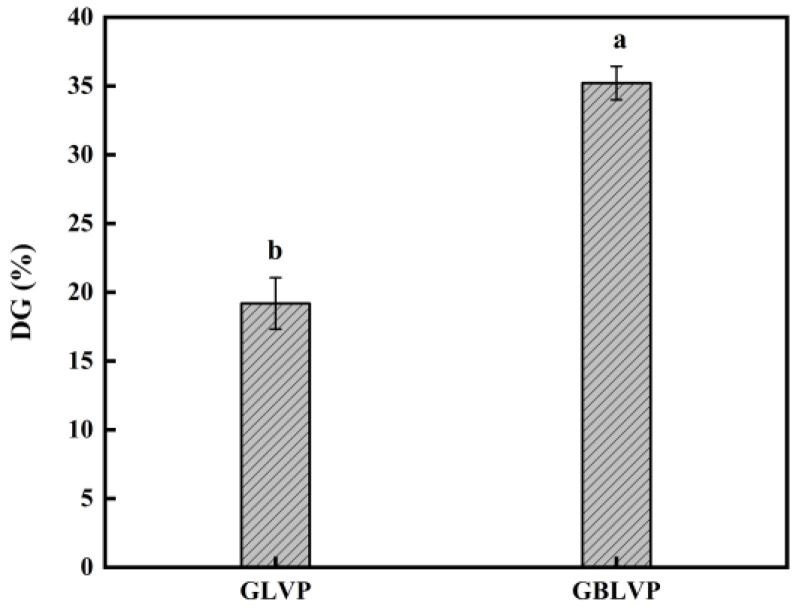
The degree of grafting (DG) of LVP under different treatments. Different letters in each column represent significant differences (*p* < 0.05).

The amount of free amino groups in proteins varies depending on the glycosylation process, thus indirectly indicating the extent of the glycosylation reaction [27]. The DG of GBLVP was significantly (*p* < 0.05) higher than that of GLVP, reaching 35.21% (Figure 2), providing evidence that the ball-milling treatment effectively enhanced the glycosylation reaction. When proteins are ball-milled, protein particle size will change and their particle size may reduce under mechanical forces [20]. As a result, ball-milled proteins might expose more glycosylation sites, which increases the contact area for the reaction and facilitates the glycosylation reaction.

### 3.2. Morphology Analysis of LVP after Different Treatments

#### 3.2.1. Microstructure Analysis

**Figure 3:**
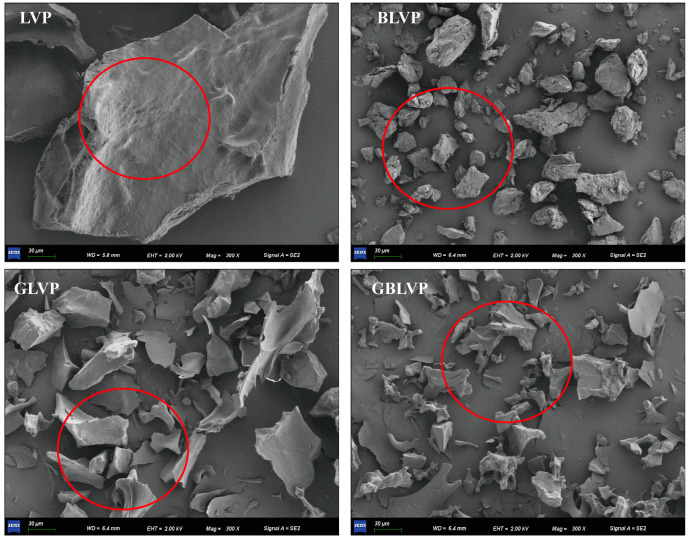
SEM images of LVP under different treatments (300×).

As shown in Figure 3, LVP mainly exhibited irregular granularity with large particle size and a smooth surface without any holes. After the ball-milling treatment, the large protein particles in BLVP were broken, resulting in smaller and uniformly distributed particles. However, after the glycosylation treatment, both GLVP and GBLVP showed a homogeneous and loose lamellar structure. These structural changes were attributed to the covalent binding between proteins and xylose molecules, which stretched the protein structure, increased surface hydrophilic groups of LVP and facilitated the outward diffusion of the conjugates [28]. In addition, GBLVP had a more uniform and sparser lamellar structure than GLVP, which could potentially be due to the mechanical impact of ball-milling. This process facilitated the dissociation of large-particle proteins in LVP and reduced their size. In conclusion, the lamellar structure observed in both GLVP and GBLVP may have been caused by the reduction of protein aggregation, which in turn enhanced their solubility and dispersion in a solution.

#### 3.2.2. Particle Size

**Figure 4:**
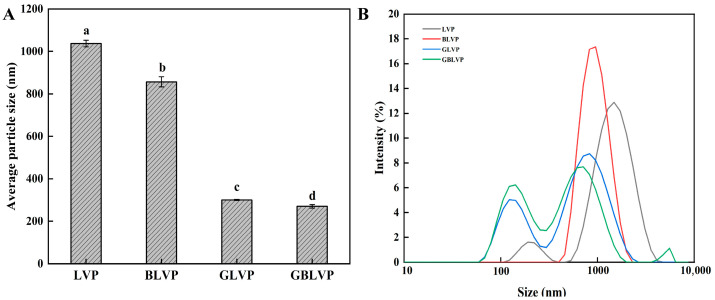
Average particle size (**A**) and particle size distribution (**B**) of LVP under different treatments. Different letters in each column represent significant differences (*p* < 0.05).

Smaller protein particles have a higher effective contact area of the particles with the solvent, thereby having higher bioavailability [29]. Figure 4A shows that the average particle size of the samples in different treatment groups decreased in the following order: LVP > BLVP > GLVP > GBLVP. The particle size distribution of LVP exhibited a peak at 1483.89 nm, while that of BLVP, GLVP and GBLVP shifted towards smaller sizes (Figure 4B). Among all the samples, GLVP and GBLVP had the biggest shift, suggesting that glycosylation could significantly (*p* < 0.05) decrease the particle size of LVP. This observation could be attributed to the hydroxyl groups in xylose, which not only enhanced the spatial repulsion between LVP molecules, but also inhibited their binding tendency [12]. The GBLVP had the smallest size. The friction and crowding forces during ball-milling pretreatment reduced the protein particle size [30] and promoted the binding between xylose molecules, thereby preventing the aggregation of proteins.

### 3.3. Effects of Different Treatments on Structural Characterizations of LVP 

#### 3.3.1. Secondary Structure

**Figure 5:**
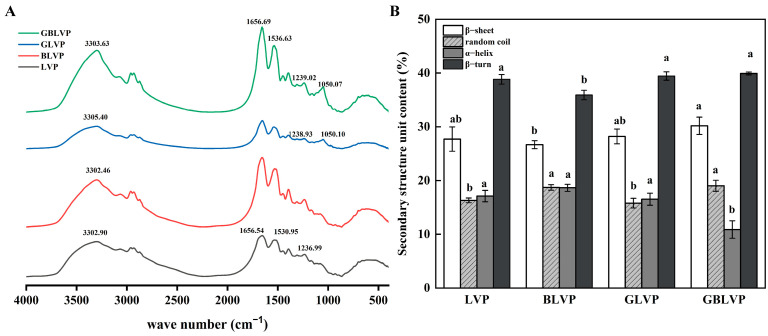
FTIR spectra (**A**) and secondary structure composition (**B**) of LVP under different treatments. Different letters in each column represent significant differences (*p* < 0.05).

The FTIR spectra are shown in Figure 5A to reflect the molecule structure changes of the samples after different treatments. The characteristic absorption peaks of GBLVP and GLVP at around 1050.10 cm^−1^ indicated the successful glycosylation of LVP with xylose. The amide III bands of GLVP and GBLVP were shifted from 1236.99 cm^−1^ to 1238.93 cm^−1^ and 1239.02 cm^−1^, respectively. These shifts indicated the stretching vibration of C-N and confirmed the successful grafting of the xylose carbonyl group to the amino group of LVP [12].

The shift in the amide I band of GBLVP from 1656.54 cm^−1^ to 1656.69 cm^−1^ suggested the Schiff bases were formed during glycosylation [31]. The amide II band of GBLVP, as a result of N-H bending vibration, was shifted from 1530.95 cm^−1^ to 1536.63 cm^−1^ for LVP. This shift indicated the involvement of N-H bonds in the reaction. A series of peaks were observed in the carbohydrate characterization region (1180–953 cm^−1^) due to the vibration of bonds, like C-O, C-C and C-H [32]. In the above region, the absorption intensity of GBLVP was higher than that of LVP, which was due to the enhancement of the absorption intensity caused by the binding between LVP and xylose [33]. Particularly, the absorption intensity of the co-treated GBLVP surpassed that of the conventionally heated GLVP, indicating that the ball-milling treatment could greatly facilitate the grafting reaction, in agreement with the DG result (Figure 2). The absorption peaks of GLVP and GBLVP shifted from 3302.90 cm^−1^ to higher wave numbers, indicating that the hydrogen bonds were broken during glycosylation and the structure of LVP became loose. 

As shown in Figure 5B, the GBLVP had lower α-helix (10.88%) and higher β-sheet (30.19%) and β-turn (39.92%) contents compared to those of non-glycosylated LVP. The GLVP had a lower α-helix (16.54%) content, but higher β-turn (39.45%) and β-sheet (28.21%) contents compared to those of non-glycosylated LVP. The decrease in α-helix content might be due to the depletion of ε-amino groups located in the α-helix during glycosylation of xylose [34]. Significantly, the α-helix content of GBLVP was lower than that of GLVP, indicating there was a higher content of sugar molecules binding to GBLVP. These findings suggested that the secondary structure of LVP was altered during treatment and ball milling effectively enhanced the glycosylation. The decrease in the α-helix content and the increase in the β-turn content implied a more relaxed secondary structure of the LVP-dextran conjugate. 

#### 3.3.2. Surface Hydrophobicity

**Figure 6:**
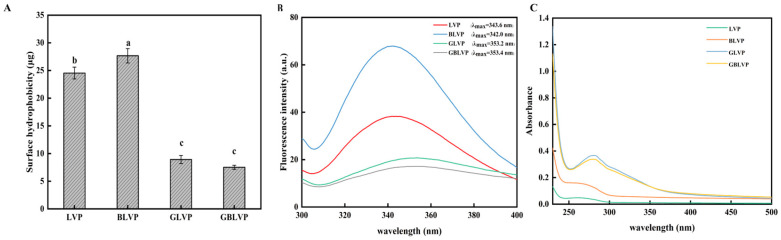
Surface hydrophobicity (**A**), fluorescence spectra (**B**) and UV absorption spectra (**C**) of LVP under different treatments. Different letters in each column represent significant difference (*p* < 0.05).

H_0_ indicates the number of hydrophobic groups exposed by the protein molecules. It is supposed as an important force to maintain the tertiary structure of proteins [35]. As shown in Figure 6A, the H_0_ values of GLVP and GBLVP were lower than those of LVP and BLVP. The H_0_ of GBLVP was the lowest, which was consistent with the result of the DG of glycosylation (Figure 2). This reduction declared that the introduction of sugar molecules could increase the number of hydrophilic groups [36]. The combined ball-milling treatment would further promote the exposure of LVP molecules, leading to a more covalent binding between hydroxyl-containing xylose and LVP, thus significantly increasing the hydrophilicity of the molecules. Additionally, hydrophobic groups might have rearranged as the protein molecules unfolded and refocused during the reaction phase, which subsequently formed the internal hydrophobic regions and decreased values of H_0_ [37]. In conclusion, both the glycosylation and the combined treatments can improve the hydrophilicity of proteins.

#### 3.3.3. Intrinsic Fluorescence Emission Spectra 

Fluorescence spectra of proteins are utilized for characterizing changes in the tertiary structure of proteins [38]. As shown in Figure 6B, the highest fluorescence intensity was observed in BLVP, which suggested the mechanical influence of the ball-milling treatment resulted in the unfolding of the LVP structure and exposed more tryptophan residues. However, the fluorescence intensity of GLVP and GBLVP decreased after xylose glycosylation. It could be explained by the gradual unfolding of the protein during glycosylation, which, in turn, overexposed the aromatic amino group to hydrophilic solvent, thereby producing a fluorescence quenching. On the other hand, it was also possible that xylose produced a spatial site-blocking effect that reduced the fluorescence intensity [39]. Furthermore, the fluorescence intensity of GBLVP was lower than that of GLVP, which was consistent with the highest degree of glycosylation (Figure 2). A similar observation has been reported in a previous study [40]. In addition, glycosylation caused the peaks of GLVP and GBLVP to be red-shifted, specifically from 343.6 nm to 353.2 nm for GLVP and from 342.0 nm to 353.4 nm for GBLVP. This result indicated that glycosylation modification could alter the conformational structure of LVP and expose its aromatic residues that were buried in a non-polar microenvironment to a polar solvent environment [3].

#### 3.3.4. Ultraviolet Absorption Spectra 

The tertiary structural changes of proteins were characterized by UV spectroscopy [41]. The UV absorbance intensities of GLVP and GBLVP were significantly higher (*p* < 0.05) compared with those of LVP and BLVP in Figure 6C. This could be attributed to the fact that glycosylation could cause the peptide chain in the proteins to become extended and undergo unfolding, in turn exposing internal tryptophan and tyrosine and increasing the corresponding absorption peaks [42]. The maximum UV absorption peak of GBLVP was slightly lower than that of GLVP, indicating that the ball-milled pretreated LVP incorporated more xylose. The interaction between xylose and LVP masked a small portion of the chromophore exposed during glycosylation. The absorption peaks of both GLVP and GBLVP experienced a red shift, indicating a change in the polar state of the microenvironment of residues such as tryptophan and tyrosine [43]. The blue shift in the absorption peak indicated the break of peptide bonds, thereby exposing more hydrophobic groups, while the red shift had the opposite effect. Therefore, the red shift in the absorption peaks of GLVP and GBLVP could explain the decrease in surface hydrophobicity observed after the glycosylation.

### 3.4. Effects of Different Treatments on Functional Properties of LVP 

#### 3.4.1. Solubility 

**Figure 7:**
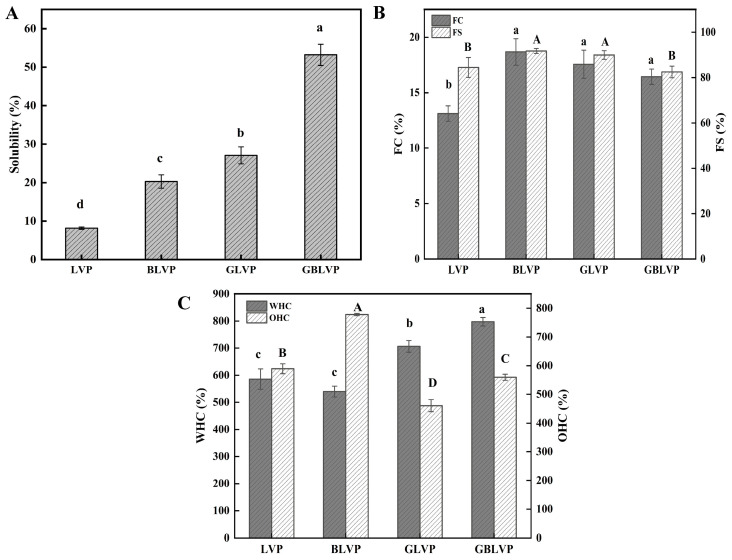
Solubility (**A**), FC and FS (**B**) and WHC and OHC (**C**) of LVP under different treatments. Different letters in each column represent significant differences (*p* < 0.05).

Solubility plays an important role in other functional properties. Figure 7A shows that the solubility of LVP increased after either ball-milling treatment or glycosylation, and that of GBLVP was the highest. The introduction of sugar chains during glycosylation buried the hydrophobic residues of proteins, while the presence of hydrophilic hydroxyl groups enhanced their hydrophilicity (Figure 6A). Additionally, the ball-milling treatment facilitated the glycosylation reaction, resulting in the introduction of more sugar chains, in turn, further improving solubility.

#### 3.4.2. FC and FS 

FC indicates the ability of the protein solution to form foam after oscillation or churning. As shown in Figure 7B, the FC values of GLVP and GBLVP increased to 17.56% and 16.44%, respectively. The increase in FC might be due to the increase in their solubility (Figure 7A), which promoted the diffusion and stretching of the proteins in the gas-liquid interface. Additionally, the hydrophilic hydroxyl groups were increased by the presence of sugar in the protein, causing the structure of proteins to become softer and looser, thus accelerating their adsorption of proteins in the gas-liquid interface [44]. However, there was no significant difference between GBLVP and BLVP or GLVP, indicating that the combined treatment did not further improve the FC of LVP.

Both GLVP and GBLVP showed a slightly decreased FS compared to BLVP, with GBLVP having the lowest FS (82.49%) (Figure 7B). A possible explanation for this was that the ball-milling treatment promoted the glycosylation reaction, but an excessive amount of hydroxyl groups (-OH) were induced at the same time. The presence of these additional hydroxyl groups then increased the electrostatic attraction and interfacial tension between protein molecules. However, the excessive increase in interfacial tension ultimately resulted in a decrease in FS [12].

#### 3.4.3. WHC and OHC 

WHC and OHC refer to the capacity of proteins to hold water and fat, respectively. Figure 7C shows the WHC of BLVP decreased while its OHC increased following ball-milling treatment. This observation was related to the exposure of hydrophobic groups (Figure 6A). Hydrophobic groups enhanced the binding affinity of the proteins with oil, while decreasing their adsorption capacity with water. Significantly, the WHC of GLVP and GBLVP increased after glycosylation treatment, and the highest WHC (797.33%) was observed in GBLVP. We can infer that the addition of sugar caused an increase in hydrophilic hydroxyl groups, resulting in an increase in WHC. Glycosylation also exposed more peptide bonds and polar side chains of proteins. These changes promoted intermolecular interactions and improved the intermolecular hydration of the protein [45]. 

From Figure 7C, we also found that the OHC of the GLVP and GBLVP decreased to 460.67% and 560.00% compared to that of LVP and BLVP, respectively. However, the OHC of GBLVP was higher than that of GLVP. These changes could also be related to the reduction in H_0_ caused by glycosylation (Figure 6A), which, in turn, caused the reduction in the adsorption of oil by protein. In addition, GBLVP showed smaller particle sizes compared to GLVP (Figure 4A) to display an increased specific surface area. Therefore, GBLVP exhibited a relatively stronger adsorption capacity and an elevated OHC as compared to GLVP.

### 3.5. Effects of Different Treatments on In Vitro Digestibility and Allergenicity of LVP 

#### 3.5.1. In Vitro Digestibility 

**Figure 8:**
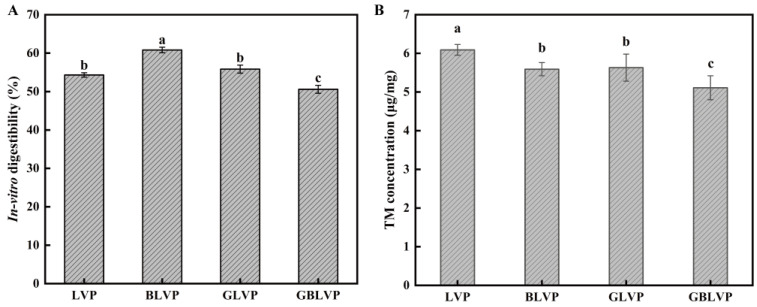
Vitro digestibility (**A**) and TM content (**B**) of LVP under different treatments. Different letters in each column represent significant differences (*p* < 0.05).

Digestibility is an essential indicator for evaluating protein quality. As shown in Figure 8A, the digestibility of BLVP was the highest compared to others, indicating the ball-milling treatment alone would significantly improve the digestibility of LVP. This could be explained by its smaller protein particle size achieved by the ball-milling, which promoted the enzyme-protein binding. However, the glycosylated GLVP showed no significant change (*p* > 0.05) in digestibility. The combined treatment even decreased the digestibility of GBLVP to 50.59%, which was the lowest among all samples. It seemed that glycosylation may have had a detrimental impact on protein digestibility by modifying the protein conformation or obstructing enzyme cleavage sites [46]. Lysine usually serves as a potential site for glycosylation and trypsin cleavage in proteins [47]. Meanwhile, according to the Figure 2, GBLVP was significantly more grafted than GLVP. Herein, after the ball milling, the protein structure unfolded and more xylose was grafted to LVP. As a consequence, more lysine was consumed during glycosylation, leading to a reduction in the available enzymatic sites. Additionally, xylose induced the cross-linking of protein molecules and inhibited protein hydrolysis. Consequently, GBLVP had the lowest digestibility.

#### 3.5.2. Allergenicity 

TM is the main cause of allergic reaction to shrimp [48].The TM contents in GLVP and GBLVP were lower compared to that in LVP (Figure 8B). It is well documented that in addition to lysine and histidine, which contain free ε-amino acids, arginine also plays a crucial role in the glycosylation of proteins [49]. However, these three amino acids are also situated within the epitope of TM [50]. Therefore, the reduction in allergenicity could be attributed to glycosylation, which affects lysine, arginine and histidine. Glycosylation could mask the allergenic epitopes, preventing the binding of antigens and antibodies, thereby diminishing the binding capacity of LVP with IgG and IgE. A similar outcome was found in the study by Liu et al. [51]. An additional factor is that glycosylation might disrupt both conformational and linear epitopes [52]. Among all the samples, GBLVP showed the highest reduction in TM content, from 6.09 μg/mg to 5.11 μg/mg, with an allergenicity reduction rate of 16.09%. This might be explained by the fact that the antigenic epitopes of TM were destroyed during ball milling by the application of high-frequency oscillatory pressure. Consequently, the combined treatment further diminished the binding capacity of allergens and antibodies, which would potentially reduce the risk of allergic reaction.

## 4. Conclusions

This study focused on elucidating the effects on the structure and functional properties of LVP treated by ball-milling treatment, glycosylation alone and combined treatment. The FTIR results showed the successful grafting of xylose onto LVP. The DG values of conjugates were greatly raised by the ball-milling pretreatment, indicating that the pretreatment is superior to the conventional wet heat method. Meanwhile, GBLVP had a sparser lamellar structure, a smaller particle size and lower surface hydrophobicity. The changes in its secondary structures and aromatic amino acid microenvironment confirmed that the ball-milling treatment combined with glycosylation altered its protein structure. These structural changes further improved the solubility, FC and WHC of LVP and reduced its allergenicity. Thus, ball-milling treatment could facilitate the glycosylation reaction of proteins and improve functional properties of conjugates. This research is expected to provide more potential LVP sources with good functional properties for the food industry.

## Data Availability

The original contributions presented in the study are included in the article, further inquiries can be directed to the corresponding author.
